# Statins and Pacing-Induced Atrial Myopathy: Unraveling Their Role in Atrioventricular Block Patients with Permanent Pacemakers

**DOI:** 10.7150/ijms.113087

**Published:** 2025-07-19

**Authors:** Yu-Sheng Lin, Wan-Chun Ho, Meng-Hung Lin, Ting-Yu Kuo, Chang-Min Chung, Pei-Chun Yen, Yung-Lung Chen, Huang-Chung Chen, Wei-Chieh Lee

**Affiliations:** 1 Division of Cardiology, Chang Gung Memorial Hospital, Chiayi, Taiwan.; 2 Health Information and Epidemiology Laboratory, Chang Gung Memorial Hospital, Chiayi Branch, Taiwan.; 3 Division of Cardiology, Department of Internal Medicine, Kaohsiung Chang Gung Memorial Hospital, Chang Gung University College of Medicine, Kaohsiung, Taiwan.; 4 School of Medicine, College of Medicine, National Sun Yat-sen University, Taiwan.; 5 Division of Cardiology, Department of Internal Medicine, Chi Mei Medical Center, Tainan, Taiwan.

**Keywords:** atrioventricular block, statin, atrial myopathy

## Abstract

**Background:** Left atrial (LA) myopathy from interventricular dyssynchrony often precedes atrial fibrillation (AF) in atrioventricular block (AVB) patients with frequent right ventricular (RV) pacing, but the role of statin therapy in preventing LA myopathy and associated arrhythmias remains debated.

**Objectives:** This study investigated the mechanisms of LA myopathy and the clinical outcomes of statin therapy in AVB patients with permanent pacemakers (PPMs).

**Methods:** The study comprised an animal, cell, and clinical cohort study. In the animal study, 12 Lanyu miniature pigs were divided into sham control, RV pacing, and RV pacing plus atorvastatin groups to assess cardiac effects over six months. The cellular study used HL-1 atrial myocytes to evaluate fibrosis and protein expression after cyclic stretching. The clinical study included 2338 AVB patients with PPM, comparing statin and non-statin groups for AF incidence and outcomes using inverse probability of treatment weighting (IPTW).

**Results:** In the animal model, atorvastatin did not affect LA size, function, or fibrosis. The cellular study showed increased fibrosis markers in both stretch and stretch plus atorvastatin groups compared to controls. In the PPM cohort, statin use did not significantly impact LA size, AF incidence (p=0.731), or CV mortality (p=0.129) over five years, but it was associated with significantly lower all-cause mortality (p=0.004) after IPTW adjustment.

**Conclusion:** Statins do not appear to improve LA myopathy or reduce the incidence of associated atrial arrhythmias in the AVB population with PPMs.

## Introduction

Atrial fibrillation (AF) is the most prevalent cardiac arrhythmia encountered in clinical practice, with its occurrence rising significantly with age; it affects 5% of individuals over 65 and 10% of those over 80. 1 As society ages, the demand for permanent pacemakers (PPMs) has steadily increased among patients with bradycardia. 2 Pacemaker with right ventricular (RV) pacing can lead to interventricular dyssynchrony, which is a significant issue linked to AF and an increased long-term risk of stroke. 3 Interventricular dyssynchrony caused by RV pacing may contribute to the development of atrial myopathy, such as atrial inflammation, atrial fibrosis, increased atrial oxidative stress and atrial enlargement, which can lead to the development of AF. 4 A significant link between inflammation, oxidative stress, and the onset, recurrence, and persistence of AF has been demonstrated in the clinical scenario between cardiac surgery and AF because of structural changes in the atria driven by active inflammatory process following the surgical procedure. 5, 6 Consequently, treatments aimed at reducing atrial inflammation and fibrosis may lower the risk of AF, and statins played a potential role in reducing inflammatory process. 7-10

Statins, formally known as 3-hydroxy-3-methyl coenzyme A reductase inhibitors, are the most widely used lipid-lowering agents. Statins are frequently classified as part of upstream therapies for AF, a category that also includes agents targeting the renin-angiotensin system. 11 For both primary and secondary prevention of AF, statins offer varying degrees of benefit in reducing AF recurrence. 12-14 The use of statins may reduce the incidence of AF, particularly in patients with sick sinus syndrome and an enlarged atrium. 15-17 The randomized study demonstrated the effectiveness of atorvastatin in preventing significant AF and LA enlargement in patients with PPMs. 15 Therefore, the potential benefit of statin in preventing fibrosis and atrial myopathy is worth considering and warrants further exploration. The involvement of the Glycogen synthase kinase-3 beta (GSK-3β) signaling pathway is critical in the pathogenesis of atrial myopathy, particularly through its regulatory effects on fibrosis, atrial remodeling, and related cellular processes, as supported by its roles in upstream and downstream molecular interactions influencing fibrotic and metabolic pathways. 18, 19 However, the impact of statins on PPM-related AF in patients with atrioventricular block (AVB) remains a subject of debate.

Accordingly, this study aimed to investigate the effect of statins on PPM-related AF in the context of AVB, bridging findings from bench research to clinical practice.

## Methods

### Animal model

In our study, we used 12 male Lanyu miniature pigs, randomly assigned to one of three groups: the sham control group (n = 4), the RV pacing group (n = 4), and the RV pacing plus atorvastatin (RVA) group (n = 4) (Supplemental Figure 1). All pigs received care in accordance with the Guide for the Care and Use of Laboratory Animals, published by Taiwan's National Institutes of Health. The animal procedures were approved by the Institutional Animal Care and Use Committee (IACUC:2018030801) at Chang Gung Memorial Hospital. Each pig underwent a surgical procedure to place pacing leads, with the atrial lead positioned at the right atrial appendage and the ventricular lead at the high septum of the RV. In addition, complete AVB was induced by radiofrequency ablation of the AV node (Supplemental Figure 2). The generator was then connected to the pacing leads and set to VDD (ventricular pacing and dual sensing) mode for pigs in both the RV and RVA groups. These procedures were conducted on pigs aged 8-10 months, and all pigs were sacrificed at the 6-month follow-up post-surgery. This pacing duration was selected based on our previous study, which demonstrated observable changes in atrial tissue over this period 4. Throughout the study, all experimental pigs were fed a regular diet, with the RVA group receiving atorvastatin (40mg/day) in their diet starting two weeks before surgery and continuing until the sacrifice date. Atorvastatin 40 mg/day was selected based on prior studies showing effective lipid-lowering and pleiotropic effects in large animal models, including our previous study. 20, 21. Transthoracic echocardiography was performed under sedation on two occasions: once within one week before surgery and again before sacrifice. Left atrial ejection fraction (LAEF), and LA end-systolic area and volume (ESA and ESV), as well as end-diastolic area and volume (EDA and EDV), were measured. Further details on experimental procedures, including Masson's trichrome staining, and Western blotting for collagen III and GSK expression measurement, are provided in the supplemental methods section.

### Cellular model

HL-1 atrial myocytes were subjected to 10% uniaxial cyclic stretching at 1Hz for 24hr to investigate the effects of mechanical stress and atorvastatin on fibrosis and downstream protein pathways. The cells were divided into four groups: a control group (n = 4), a stretch group (n = 4) that underwent 10% uniaxial cyclic stretch at 1 Hz for 24 hours, a stretch group treated with 1 μM atorvastatin (Stretch-A, n = 4) for 24 hours, and a group treated with 40 mM Lithium Chloride (LiCl) for one hour (Supplemental Figure 3). Additionally, Western blot analysis was performed to examine the levels of collagen III (1:1000 dilution; Proteintech, TX, USA) and the phosphorylation status of GSK3β and p-GSK3β (Ser9) (1:1000 dilution; Cell Signaling, MA, USA), normalized to GAPDH (1:1000 dilution; Proteintech, TX, USA), to explore the involvement of the GSK3β pathway in fibrosis.

### Clinical cohort study

Between January 1, 2001, and December 31, 2022, a total of 4,705 patients who underwent pacemaker implantation for Mobitz type II or complete AVB were identified from the Chang Gung Research Database (CGRD) in Taiwan, which includes data from four tertiary care medical centers and three major teaching hospitals (Supplemental Figure 4). A similar cohort study was conducted to investigate pacing-induced cardiomyopathy, involving different participants and varying enrollment durations 20. Patients' diagnoses, laboratory results, imaging data, and detailed chart records were thoroughly evaluated in this study. Some patients were excluded, such as those under 18 years of age, those with a history of malignancy, AF/flutter, conduction system pacing, or myocardial infarction, and those who began statin therapy more than six months after pacemaker implantation. This selection process resulted in 2,338 eligible patients, with 304 patients in the statin group and 2,034 patients in the non-statin group. The long-term use of statins, such as atorvastatin, rosuvastatin, pitavastatin, fluvastatin, pravastatin, and simvastatin, was defined as continuous statin therapy for at least three months following the pacemaker implantation. The primary outcome of interest was the newly development of AF. Secondary outcomes included all-cause mortality and cardiovascular (CV) mortality. CV mortality was defined based on standardized criteria, including death due to acute myocardial infarction, sudden cardiac death, heart failure, stroke, and other CV causes. Additionally, echocardiographic data, including LA dimension, left ventricular ejection fraction (LVEF), and left ventricular end-diastolic diameter (LVEDD), were assessed during the follow-up period, using M-mode or 2D Simpson method in transthoracic echocardiography. The study was conducted with the approval of the Institutional Review Board of Chang Gung Memorial Hospital (IRB: 202202243B0).

### Statistics

All continuous variables from laboratory results are expressed as mean ± standard deviation or standard error of the mean. For comparisons involving three or more groups, one-way ANOVA was used, while the Mann-Whitney Test was employed for comparisons between two groups. In the clinical cohort study, we used inverse probability of treatment weighting (IPTW) based on propensity scores to ensure comparability between the statin and non-statin groups when evaluating outcomes. The propensity scores were generated through multivariable logistic regression, with the study group regressed on age, gender, comorbidities, and medications (as detailed in Table S1). Stabilized weights were applied to minimize the effect of extreme propensity scores. The balance of covariate distribution between groups was assessed using the absolute standardized mean difference, with a threshold of <0.1 indicating a minimal difference. In terms of echocardiographic parameters, LA, LVEF, and LVEDD were compared between the statin and non-statin groups at four different time points using independent sample t-tests. To adjust for multiple testing, the Bonferroni correction was applied, resulting in an adjusted significance level of 0.0125. Time-to-event outcomes, including AF incidence, CV mortality, and all-cause mortality, were analyzed using Cox proportional hazards models, with results expressed as hazard ratios (HRs) with 95% confidence intervals (CIs). The incidence of AF, CV mortality, and all-cause mortality between the statin and non-statin groups was compared using the Kaplan-Meier method, and the differences between groups were assessed by the log-rank test. A two-sided P value of <0.05 was considered statistically significant. All statistical analyses were conducted using SAS Version 9.4 (SAS Institute, Cary, NC, USA).

## Results

### Temporal changes in LA structural and functional parameters: a comparison of RV pacing, RV pacing plus atorvastatin, and controls

**Figure 1:**
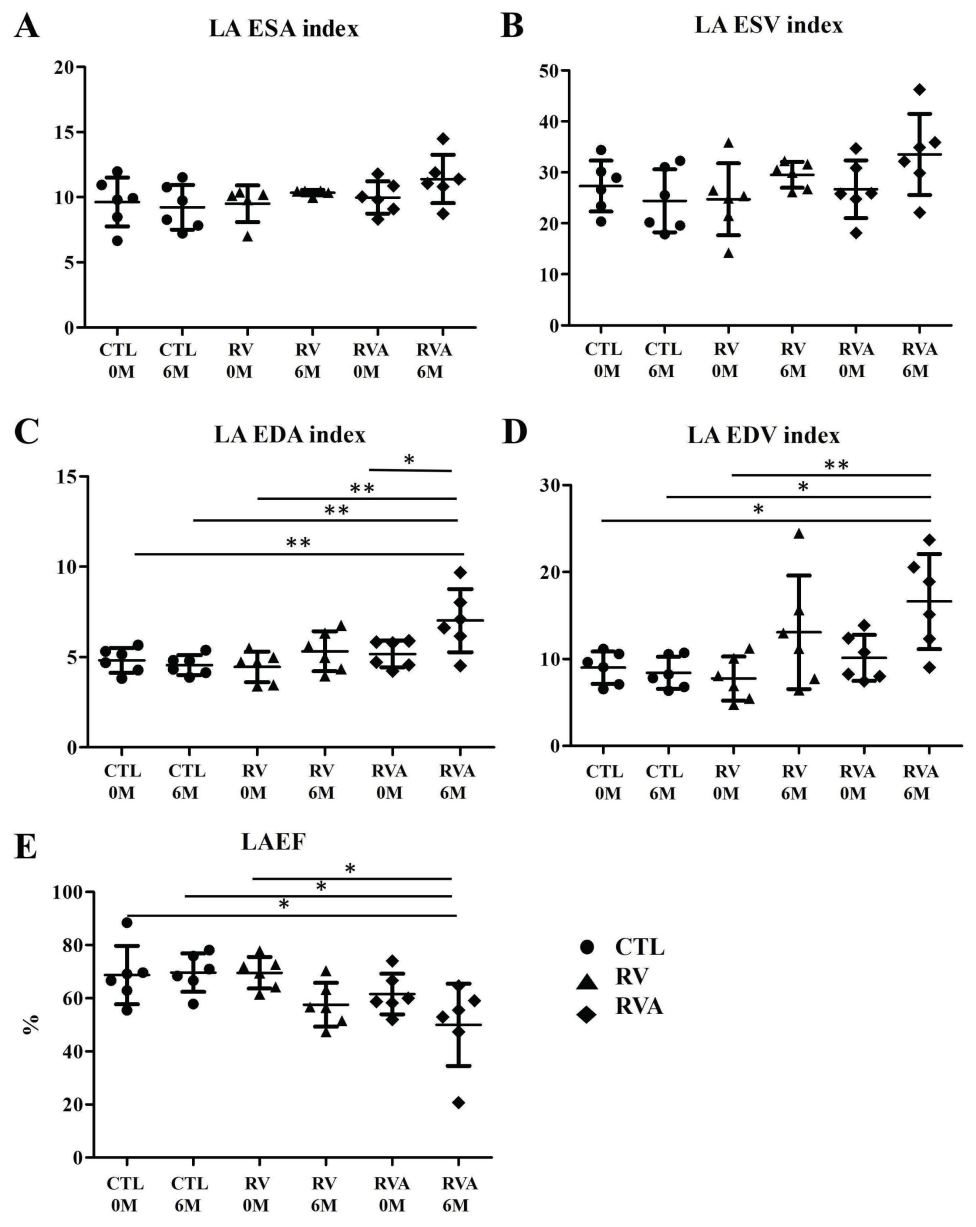
Changes in left atrial (LA) structural and functional parameters over time following right ventricular (RV) pacing compared to RV pacing plus atorvastatin (RVA) and controls (CTL). (A) LA ESA (End-systolic Area) index: No significant differences are observed across the groups (CTL, RV, and RVA) at 0 months (0M) and 6 months (6M). (B) LA ESV (End-systolic Volume) index: The LA ESV index remains stable across CTL, RV, and RVA groups at both time points, without statistically significant changes. (C) LA EDA (End-diastolic Area) index: Significant increases in LA EDA are observed in the RVA group at 6M when compared to the other groups (CTL and RV) (*P < 0.05; **P < 0.01). (D) LA EDV (End-diastolic Volume) index: Significant increases in LA EDV index are observed in the RVA group at 6M compared to the other groups (*P < 0.05; **P < 0.01). (E) LAEF (Left Atrial Ejection Fraction): A significant reduction in LAEF is observed in the RVA group at 6M compared to CTL and RV (*P < 0.05). Abbreviations: CTL = Control; RV = Right Ventricular Pacing; RVA = Right Ventricular Pacing plus Atorvastatin; ESA = End-systolic Area; ESV = End-systolic Volume; EDA = End-diastolic Area; EDV = End-diastolic Volume; LAEF = Left Atrial Ejection Fraction. Statistical significance is denoted by *P < 0.05, **P < 0.01, ***P < 0.001.

We created three groups of animal models with the average of age were 6~8 month and the weights were similar between three groups before surgery (Sham control vs. RV vs. RVA; 36.68±3.49 kg vs. 35.43±2.70 kg vs. 36.43±2.87 kg, p = 0.163). There were no significant differences among the three groups in terms of LA size and function indices at baseline (Figure 1). After six months of pacing, a significant increase in the LA EDA index was observed in both the RV and RVA groups compared to the control group (Sham control vs. RV vs. RVA; 4.30±0.36 mm² vs. 5.65±0.40 mm² vs. 6.12±0.38 mm², p < 0.05). Similarly, a significant increase in the LA EDV index was seen in the RV and RVA groups (Sham control vs. RV vs. RVA; 7.48±0.53 mm^3^ vs. 12.31±1.82 mm^3^ vs. 15.4±2.52 mm^3^, p < 0.05) compared to the control group. A similar trend was also noted in LA ESA/ESD index among groups. A similar trend was also noted in LA ESA/ESD index among groups. LA ejection fraction was significantly reduced in the RV pacing group after six months compared to the control group, although no significant difference was observed between the RV and RVA groups (Sham control vs. RV vs. RVA; 67.78±4.15% vs. 56.93±6.56% vs. 56.24±3.43%, p < 0.05). These results highlight that, while atorvastatin did not significantly impact LA size and function indices, RV pacing led to an increase in LA size and a reduction in LA function over time. Additionally, there were no significant differences in heart rate among the three groups at baseline or before sacrifice, as shown in Supplemental Figure 5 (Baseline: Sham control vs. RV vs. RVA; 107.6±5.63 bpm vs. 100.5±5.68 bpm vs. 108.8±9.23 bpm; p = 0.728; before sacrifice: 106.8±13.2 bpm vs. 86.25±5.04 bpm vs. 88.75±4.87 bpm; p = 0.078). Pacing and atorvastatin treatments did not significantly alter heart rates compared to controls.

### Extent of left atrial fibrosis in control, RV pacing, and RV pacing plus atorvastatin groups

**Figure 2:**
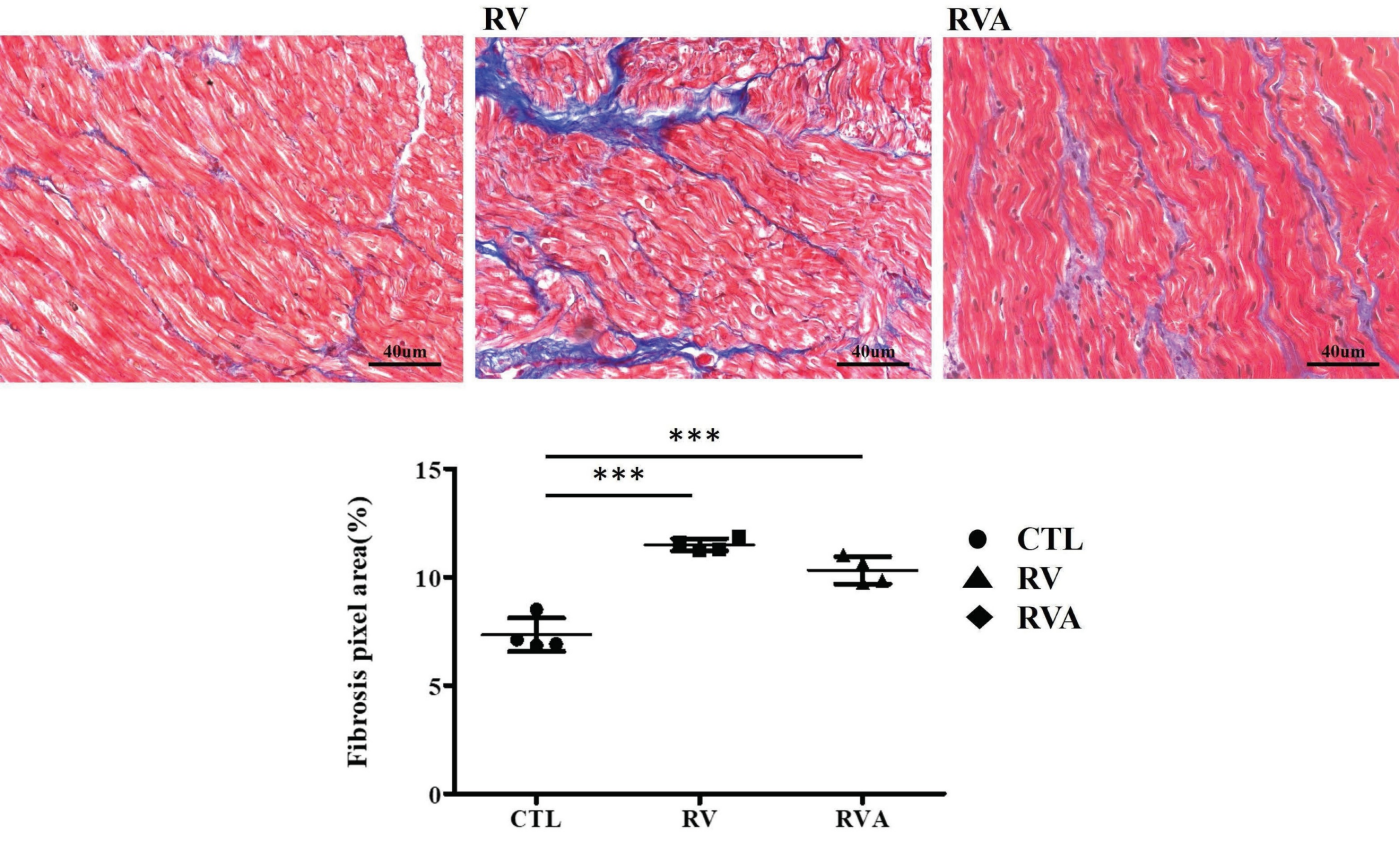
Analysis of myocardial fibrosis in control, right ventricular (RV) pacing, and RV pacing plus atorvastatin (RVA) groups. Representative Masson's trichrome staining images of left atrial tissue sections show fibrosis (blue staining) in the CTL, RV, and RVA groups. Increased fibrosis is observed in the RV and RVA groups compared to the control. The bar graph below quantifies fibrosis area (%) by pixel analysis, indicating a significant increase in fibrosis in both RV and RVA groups compared to CTL. No significant difference is observed between RV and RVA groups. Abbreviations: CTL = Control; RV = Right Ventricular Pacing; RVA = Right Ventricular Pacing plus Atorvastatin. Statistical significance is denoted by *P < 0.05, **P < 0.01, ***P < 0.001.

According to the echocardiographic results, enlarged LA, along with impaired LA function, can be induced by RV pacing. This strongly suggests pacing-induced atrial myopathy. Fibrosis, a major component of atrial myopathy, is the focus of our analysis, specifically comparing the extent of fibrosis among the three groups. Histological images stained with Masson's trichrome reveal extensive fibrosis in both the RV pacing and RVA groups compared to the control group (Figure 2). Quantification of the fibrosis percentage shows a significant increase in the RV pacing group compared to the control group (Sham control vs. RV: 7.35±0.39% vs. 11.5±0.14%, p < 0.001). Similarly, the RVA group exhibits significantly increased fibrosis compared to the control group (Sham control vs. RVA: 7.35±0.39% vs. 10.32±0.31%, p < 0.001). No difference in fibrotic percentage was observed between the RV and RVA groups. RV pacing significantly induces fibrosis in LA tissue, and atorvastatin does not prevent it. Therefore, atorvastatin does not improve pacing-induced atrial fibrosis.

### Effects of RV pacing and atorvastatin on collagen III expression and GSK phosphorylation

**Figure 3:**
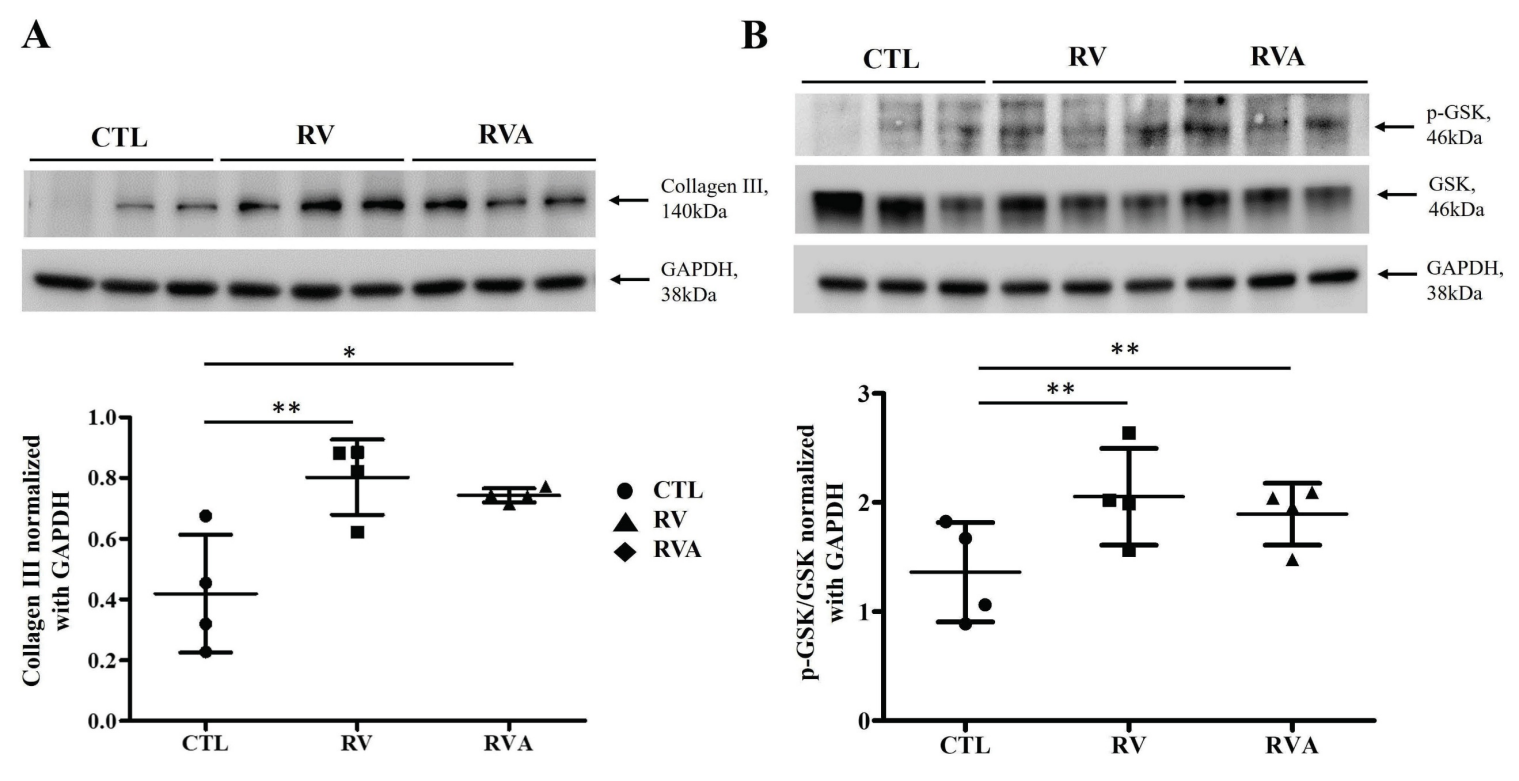
Protein expression analysis of fibrosis and glycogen synthase kinase (GSK) signaling in control, right ventricular (RV), and RV pacing plus atorvastatin (RVA) groups. (A) Western blot analysis of collagen III (140 kDa) expression in the control (CTL), RV, and RVA groups. GAPDH (38 kDa) is used as a loading control. The bar graph below represents the quantification of collagen III levels normalized to GAPDH. RV pacing significantly increases collagen III expression compared to CTL (*P < 0.05, **P < 0.01), indicating enhanced fibrosis, with no significant difference between RV and RVA groups. (B) Western blot analysis of phosphorylated GSK (p-GSK, 46 kDa) and total GSK (46 kDa) in CTL, RV, and RVA groups, with GAPDH as a loading control. The bar graph below represents the quantification of p-GSK/GSK ratios normalized to GAPDH. RV pacing significantly increases p-GSK/GSK levels compared to CTL (**P < 0.01), while no significant difference is observed between RV and RVA groups. Abbreviations: CTL = Control; RV = Right Ventricular Pacing; RVA = Right Ventricular Pacing plus Atorvastatin; GSK = Glycogen Synthase Kinase; p-GSK = Phosphorylated Glycogen Synthase Kinase; GAPDH = Glyceraldehyde 3-phosphate dehydrogenase. Statistical significance is denoted by *P < 0.05, **P < 0.01, ***P < 0.001.

RV-induced atrial myopathy and atrial fibrosis were observed, and atorvastatin did not improve these changes. The GSK-3 pathway plays a crucial role in regulating various biological and cellular processes, including metabolism, apoptosis, cell proliferation, and fibrosis 18, 19. Therefore, collagen and GSK-3 expression were examined across the three groups for comparison. The quantification of collagen III and GSK phosphorylation (p-GSK) among the control, RV, and RVA groups was analyzed (Figure 3). Western blot results for collagen III show a significant increase in collagen III expression in both the RV and RVA groups compared to the control group (Sham control vs. RV vs. RVA: 0.35±0.10 vs. 0.74±0.78 vs. 0.71±0.03; p < 0.05 and < 0.01; respectively) (Figure 3A). Similarly, Western blot results for p-GSK and total GSK reveal that the p-GSK/GSK ratio is significantly higher in the RV and RVA groups compared to the control (Sham control vs. RV vs. RVA: 1.12±0.09 vs. 2.13±0.17 vs. 1.89±0.14; p < 0.01) (Figure 3B). These findings suggest that RV pacing induces elevated collagen III levels and increased GSK phosphorylation, contributing to RV pacing-induced atrial myopathy with fibrosis.

### Effects of mechanical stretch and treatments with atorvastatin and LiCl on collagen III expression and GSK phosphorylation in HL-1 cells

**Figure 4:**
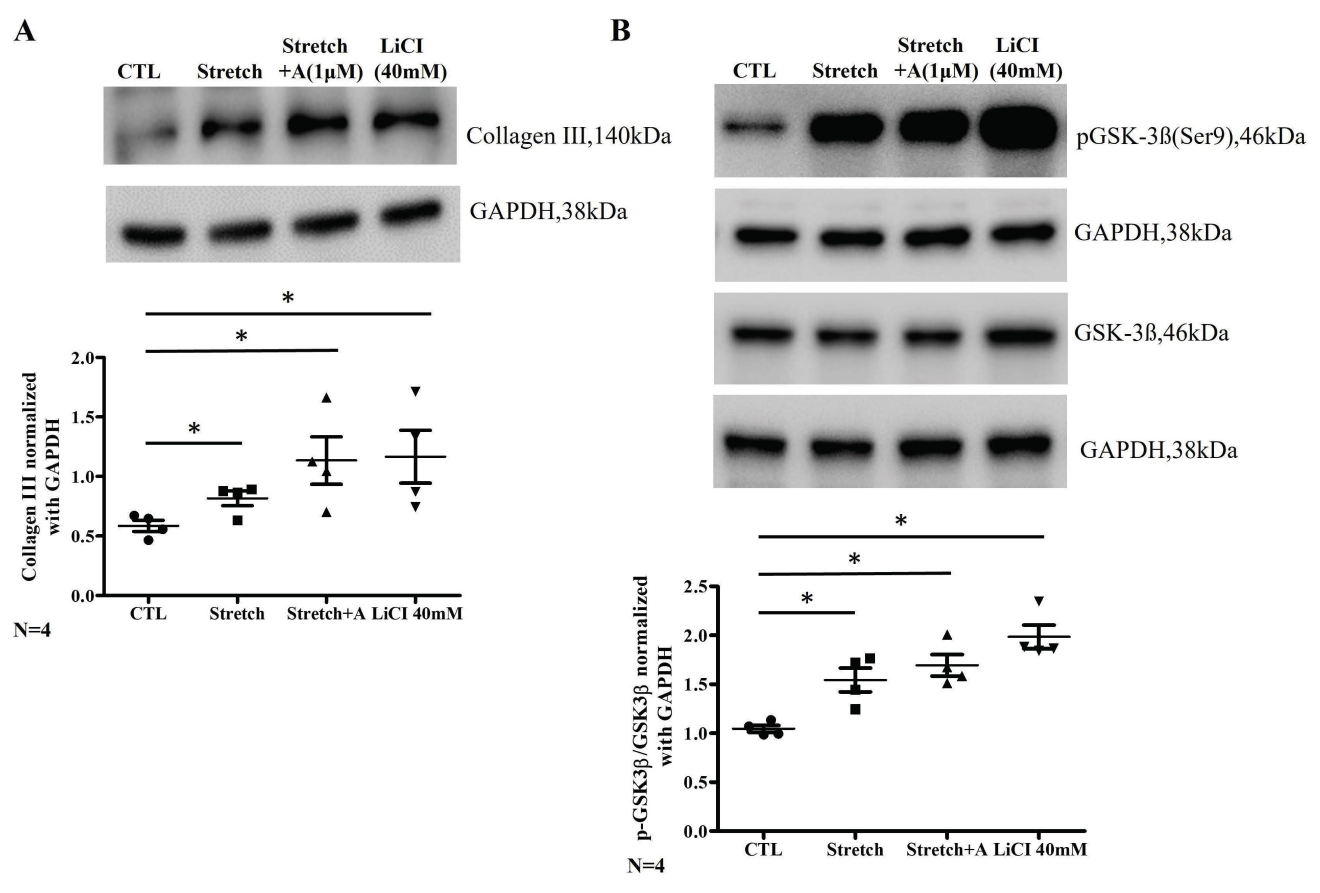
Effects of atorvastatin and LiCl on collagen III expression and GSK-3β phosphorylation in cyclically stretched HL-1 atrial myocytes. (A) Representative western blot images and quantification of collagen III protein levels (140 kDa) in control (CTL), stretch, stretch with atorvastatin (Stretch + A, 1 μM), and LiCl (40 mM) groups. Collagen III levels were normalized to GAPDH (38 kDa) as a loading control. Stretch significantly increased collagen III expression compared to control, which was not attenuated by atorvastatin or LiCl treatment. (B) Representative western blot images and quantification of phosphorylated GSK-3β at Ser9 (pGSK-3β, 46 kDa) and total GSK-3β (46 kDa) levels across the same groups. Phosphorylated GSK-3β levels were normalized to GAPDH. Stretch increased GSK-3β phosphorylation, and this effect was maintained in both the atorvastatin and LiCl treatment groups. Abbreviations: CTL = Control; Stretch-A = Stretch plus Atorvastatin; LiCl = Lithium Chloride; GSK = Glycogen Synthase Kinase; p-GSK = Phosphorylated Glycogen Synthase Kinase; GAPDH = Glyceraldehyde 3-phosphate dehydrogenase. Statistical significance is denoted by *P < 0.05, **P < 0.01, ***P < 0.001.

Due to the structural and histopathological finding of LA in animal model, a cell model subjected to mechanical stretch was used to mimic atrial stress related to interventricular dyssynchrony, and collagen III expression along with p-GSK was examined. LiCl (a GSK inhibitor) was introduced to test the presentation of fibrosis pathway. Collagen III expression is significantly increased in the stretch group compared to the control, and this increase is sustained with S+A treatment at a 1 µM concentration or LiCl treatment (CTL vs. Stretch vs. Stretch+A vs. LiCl: 0.58±0.05 vs. 0.82±0.06 vs. 1.14±0.28, 1.26±0.28; all three groups compared to CTL; p < 0.05) (Figure 4A). The p-GSK/GSK ratio is also significantly higher in the stretch group compared to the control, and this elevated ratio persists with S+A treatment or LiCl treatment (CTL vs. Stretch vs. Stretch+A vs. LiCl: 1.07±0.04 vs. 1.58±0.17 vs. 1.70±0.15, 2.03±0.16; all three groups compared to CTL; p < 0.05) (Figure 4B). These findings suggest that atorvastatin does not mitigate the effects of mechanical stretch, which promotes collagen III expression and GSK phosphorylation.

### A comparative analysis of baseline characteristics of statin and non-statin therapy in matched retrospective cohort study

In both animal and cell models, atorvastatin does not appear to improve atrial myopathy related to mechanical stretch caused by pacing-related interventricular dyssynchrony. A large clinical cohort study was conducted to assess the effects of statins on pacing-induced atrial myopathy in real-world practice. The baseline characteristics of the unweighted and IPTW cohorts compared patients on statin therapy versus non-statin therapy (Supplemental Table 1). It includes demographic variables such as age and gender, along with clinical parameters including comorbidities and medications. The table shows the absolute standardized mean differences (ASMD) for each variable, indicating the balance (ASMD<0.100) between the treatment groups post-weighting.

After applying IPTW, the incidence of heart failure admissions did not significantly differ between the statin and non-statin groups (statin vs. non-statin; 6.6% vs. 5.8%, respectively; ASMD = 0.012), nor did the incidence of AF (statin vs. non-statin; 10.5% vs. 10.6%; ASMD = 0.012). Both before and after IPTW, CV mortality rates were higher in the non-statin group compared to the statin group (Before: statin vs. non-statin; 11.5% vs. 15.2%; ASMD = 0.108; After: statin vs. non-statin; 11.5% vs. 16.7%; ASMD = 0.150). Similarly, the all-cause mortality rates were significantly higher in the non-statin group both before and after IPTW (Before: statin vs. non-statin; 37.2% vs. 49.3%; ASMD = 0.247; After: statin vs. non-statin; 37.2% vs. 54.0%; ASMD = 0.342).

### Incidence of AF and clinical outcomes between statin and non-statin groups over a 5-year follow-up period

Before and after IPTW, the HR for incidence of AF did not differ between statin and non-statin groups (Supplemental Table 2). The IPTW analysis indicated a consistent protective effect of statins, with significant reductions in CV mortality and all-cause mortality at all time points. At 5 years, the IPTW-adjusted HR for CV mortality was 0.57 (95% CI: 0.42-0.78, p < 0.001) and for all-cause mortality was 0.58 (95% CI: 0.49-0.69, p < 0.001).

**Figure 5:**
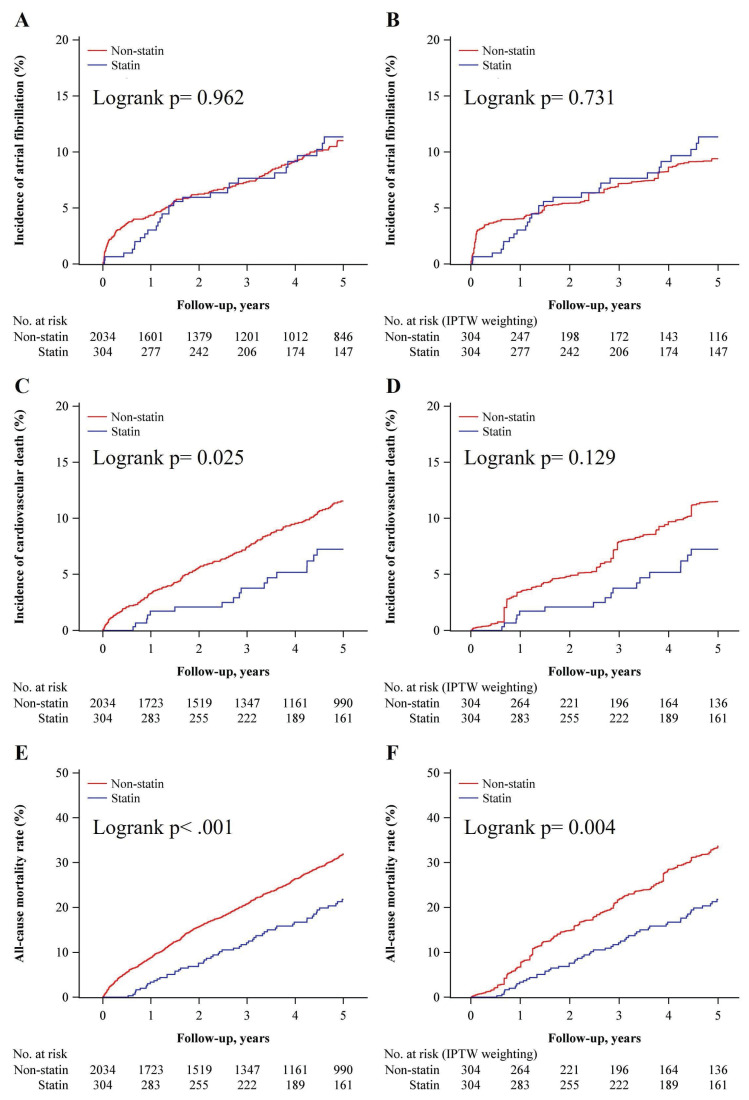
Kaplan-Meier curves comparing the incidence of atrial fibrillation, cardiovascular mortality, and all-cause mortality in patients on statins vs. non-statins. (A) Kaplan-Meier curve showing the incidence of atrial fibrillation in the non-statin group (red) and statin group (blue) over 5 years. The log-rank test shows no significant difference between groups (P = 0.962). The number of patients at risk is listed below the graph for each time point. (B) Incidence of atrial fibrillation after inverse probability of treatment weighting (IPTW) adjustment. No significant difference is observed between the statin and non-statin groups (log-rank P = 0.731). (C) Kaplan-Meier curve showing the incidence of cardiovascular mortality in the non-statin and statin groups. Statin use is associated with a significantly lower incidence of cardiovascular mortality (log-rank P = 0.025). (D) Incidence of cardiovascular mortality after IPTW adjustment. The difference between groups is no longer statistically significant (log-rank P = 0.129). (E) Kaplan-Meier curve showing all-cause mortality in the non-statin and statin groups. Statin use is associated with a significantly lower all-cause mortality rate (log-rank P < 0.001). (F) All-cause mortality after IPTW adjustment. Statin use remains associated with significantly lower all-cause mortality (log-rank P = 0.004). Abbreviations: IPTW = Inverse Probability of Treatment Weighting.

A Kaplan-Meier survival curves for the incidence of AF showed no significant difference between the groups the unweighted (Log-rank p = 0.962) and IPTW cohorts (Log-rank p = 0.731) (Figure 5A and 5B). The incidence of CV mortality showed a significant reduction in the statin group compared to the non-statin group in the unweighted analysis (Log-rank p = 0.025) (Figure 5C) but shows no significant difference with IPTW-weighting (Log-rank p = 0.129) (Figure 5D). A significantly lower all-cause mortality rate in the statin group presented with both unweighted (Log-rank p < 0.001) and IPTW-weighted (Log-rank p = 0.004) (Figure 5E and 5F). These results suggest that while statin therapy does not significantly impact the incidence of AF, it is still associated with a significant reduction in all-cause mortality over a 5-year follow-up period.

### Comparative analysis of LA dimension, LVEF, and LVEDD between statin and non-statin groups over a 5-Year follow-up period

**Figure 6:**
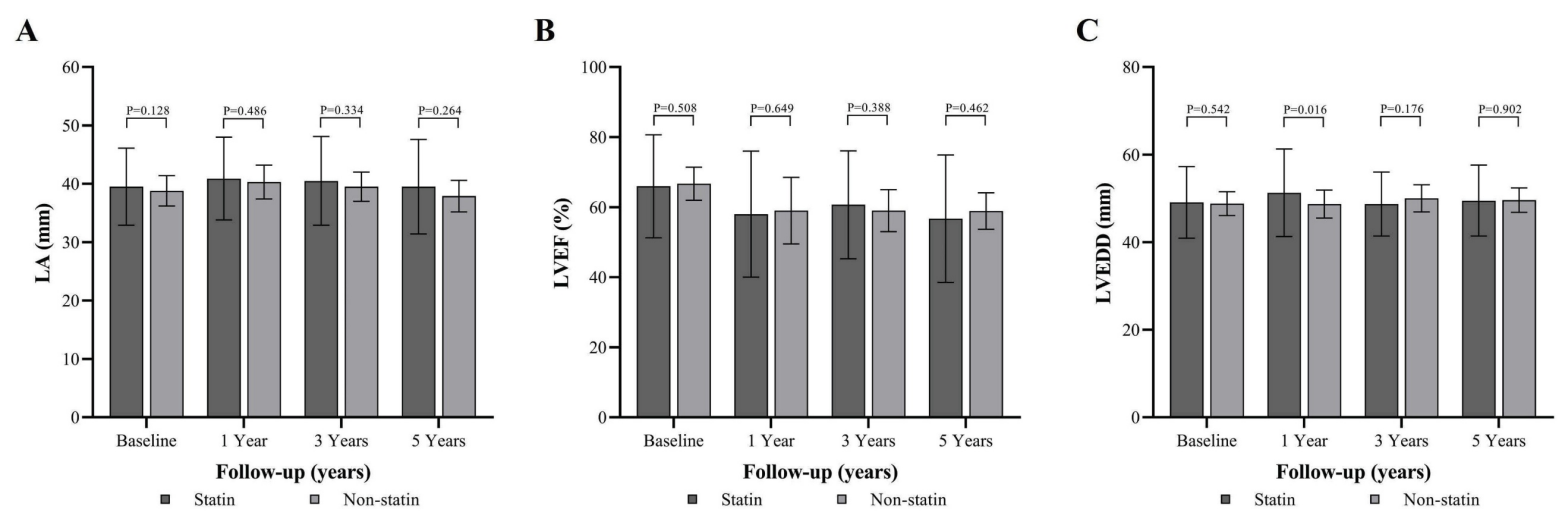
Longitudinal echocardiographic parameters comparing statin and non-statin groups over 5 years of follow-up. (A) Left atrial (LA) diameter in millimeters at baseline, 1 year, 3 years, and 5 years of follow-up in the statin (dark bars) and non-statin (light bars) groups. No significant differences are observed in LA diameter between groups at any time point (baseline: P = 0.128, 1 year: P = 0.486, 3 years: P = 0.334, 5 years: P = 0.264). (B) Left ventricular ejection fraction (LVEF, %) at baseline, 1 year, 3 years, and 5 years of follow-up. No significant differences are observed between the statin and non-statin groups in LVEF at any time point (baseline: P = 0.508, 1 year: P = 0.649, 3 years: P = 0.388, 5 years: P = 0.462). (C) Left ventricular end-diastolic diameter (LVEDD, mm) at baseline, 1 year, 3 years, and 5 years of follow-up. Statistically significant differences are observed between the statin and non-statin groups at 1 year (P = 0.016), but no significant differences are found at baseline (P = 0.542), 3 years (P = 0.176), or 5 years (P = 0.902). Abbreviations: LA = Left Atrium; LVEF = Left Ventricular Ejection Fraction; LVEDD = Left Ventricular End-Diastolic Diameter.

There was no significant difference in LA size between statin and non-statin groups at baseline, 1 year, 3 years, and 5 years ((Figure 6A and Supplemental Table 3). The LVEF indicated no significant differences between the groups at baseline, 1 year, 3 years, and 5 years (Figure 6B and Supplemental Table 3). A borderline difference was observed between the statin and non-statin groups (p = 0.016) at 1 year under the Bonferroni correction (Figure 6C and Supplemental Table 3). These results suggest that statin therapy does not have a significant impact on the LA size over a long-term follow-up period.

## Discussion

This study aimed to evaluate the impact of statin therapy on atrial myopathy and clinical outcomes in patients with AVB undergoing RV pacing, integrating findings from an animal model, a cell study, and a clinical cohort analysis. The animal study demonstrated that atorvastatin did not significantly reduce LA size or improve LA function, and significant fibrosis was still observed in both RV pacing and atorvastatin-treated groups. The cell model confirmed that mechanical stretch increased collagen III expression and GSK phosphorylation, with atorvastatin and a GSK inhibitor maintaining these elevated levels, indicating a role in fibrosis pathways. In the clinical cohort, comprising 2338 AVB patients under PPM implantation, statin use did not significantly reduce the incidence of AF after IPTW adjustments. However, statin therapy was associated with a significant reduction in all-cause mortality over a five-year follow-up period. These findings suggest that while statins may improve overall survival, they do not provide significant protection against LA atrial myopathy or prevent AF in the context of RV pacing-induced interventricular dyssynchrony, underscoring the need for alternative therapeutic strategies in this patient population.

The GSK-3 pathway plays a significant role in various biological processes and cellular processes including metabolism, apoptosis, cell proliferation, and fibrosis. 22, 23 In addition, the GSK-3 pathway is closely linked to the pathophysiology of cardiac fibrosis and pacing-induced atrial myopathy by increasing fibroblast activity and extracellular matrix production. 24, 25 Statins, such as atorvastatin and simvastatin, modulate GSK-3β activity by promoting its phosphorylation and reduces GSK-3β's role in promoting pro-inflammatory and pro-fibrotic signaling, thus contributing to the cardioprotective effects of statins. 26, 27 In our animal RV pacing model, atorvastatin therapy did not lead to a reduction in the fibrotic percentage of the LA, nor did it affect the levels of collagen and GSK. These findings suggest that atorvastatin may not effectively target the pathways involved in atrial fibrosis in this pacing-induced atrial myopathy. Similarly, in the cell model, atorvastatin therapy also failed to reduce the expression of collagen III and the level of GSK. This indicates that atorvastatin's pleiotropic effects, often seen in other cardiovascular contexts, may not extend to reducing fibrosis or modifying GSK-3β activity in the specific environment of pacing-induced cardiac stress. This highlights that atorvastatin may have limitations in addressing fibrosis at the molecular level in pacing-induced stress conditions, contrasting its known anti-fibrotic effects in other cardiovascular conditions.

The role of statin therapy in both primary and secondary prevention of AF has been extensively studied. While several longitudinal studies suggest a beneficial effect of statins in the primary prevention of AF, these findings have not been conclusively supported by large randomized clinical trials. Different statins may have varying effectiveness in managing AF in specific clinical scenarios. 28 For instance, perioperative atorvastatin has shown efficacy in both primary and secondary prevention of AF, particularly following isolated coronary artery bypass graft (CABG) surgery. 29-31 However, atorvastatin has not consistently shown reliable results, and other statin as rosuvastatin have not demonstrated a significant impact on reducing the occurrence of postoperative AF. 32-34 RV pacing-induced interventricular dyssynchrony has been shown to contribute to increased atrial myopathy and long-term stroke risk, as well as the development of atrial fibrosis in animal models. 3, 4 The use of statins to prevent pacing-induced atrial myopathy and AF has shown inconsistent results 17. Statins may be more effective in reducing the incidence of AF in patients with sick sinus syndrome but not in those with AVB. 35 RV pacing for the management of AVB is associated with continuous mechanical stress on the atria due to interventricular dyssynchrony, which can lead to mitral regurgitation and LA enlargement. 36 On the other hand, the sirtuin signaling pathway may play a crucial role in the development of atrial fibrosis, while statins may not directly influence this pathway but rather enhance the activity of sirtuin 1 (SIRT1). 37, 38 This suggests that although statins have pleiotropic effects, including anti-inflammatory and antioxidant actions, their impact on the sirtuin pathway might be indirect, primarily by boosting SIRT1 activity rather than directly modulating the pathway itself. 39 In clinical studies, different statins did not provide the similar effect on the expression of SIRT1 and only effect on the endothelial nitric oxide synthase. 40, 41 Therefore, in our cell and animal studies, the use of statins does not appear to improve atrial fibrosis caused by mechanical stress by RV pacing and stretch method. In our clinical cohort study, the use of statins also did not reduce the incidence of AF, and the size of the LA may be influenced by variations in statin dosage and the specific statins used.

### Limitations

First, the study had small number and was limited to male pigs, preventing the assessment of hormonal influences, and the pig model may not fully replicate human cardiovascular responses, potentially limiting generalizability. Second, the six-month duration of the animal study and the five-year clinical follow-up might not capture the long-term effects of statin therapy on atrial remodeling and fibrosis, as these changes may require longer periods to manifest. Third, the atorvastatin dosage of 40 mg/day was selected based on existing literature and our previous study. We acknowledge that direct comparisons to human dosing are limited due to interspecies differences in metabolism, body weight, and pharmacokinetics, which may affect drug efficacy and reduce the translational applicability of our findings. Fourth, our cellular model could not fully replicate atrial mechanics under ventricular pacing conditions, particularly concerning the frequency of mechanical stretch and uniaxial cyclic stretching with 10%, 1HZ and the short duration of 24 hours. 3D engineered heart tissues or human-induced pluripotent stem cell-derived cardiomyocytes would provide a more comprehensive understanding of the cellular responses to mechanical stress and pharmacological interventions such as statin therapy. Fifth, GSK does not comprehensively address all mechanisms of atrial fibrosis, nor does it fully explore the potential role of statins in this process. However, the GSK pathway is crucial in metabolism, apoptosis, cell proliferation, and fibrosis, and is closely linked to cardiac fibrosis and pacing-induced atrial myopathy through enhanced fibroblast activity and extracellular matrix production. In our RV pacing animal model, increased GSK phosphorylation was observed, which may be attributed to RV pacing. Sixth, variability in statin types and doses was not thoroughly addressed, limiting conclusions on dose-specific effects. Seventh, while the study focused on the GSK pathway and collagen expression, other important mechanisms, such as oxidative stress, inflammation, electrical remodeling, and ion channel dysfunction, were not fully explored, limiting a broader mechanistic understanding of the process. Lastly, the retrospective design of the clinical study introduces potential biases despite IPTW adjustments, and a prospective randomized controlled trial would mitigate selection bias, and provide stronger evidence, and allow for more controlled comparisons between statin and non-statin groups, with better control over confounding variables.

## Conclusion

Statins do not appear to improve LA myopathy or reduce the incidence of associated atrial arrhythmias in the AVB population with PPMs.

## Clinical Perspectives

1. Statin therapy and pacing-induced atrial myopathy: The study reveals that statin therapy, despite its widespread use for reducing cardiovascular risk, does not significantly prevent atrial fibrosis or improve left atrial size and function in patients with atrioventricular block (AVB) undergoing right ventricular (RV) pacing. This suggests that statin therapy may not be effective in preventing atrial remodeling in this context.

2. Atrial fibrillation prevention: Statin therapy did not reduce the incidence of atrial fibrillation (AF) or heart failure hospitalizations in patients under permanent pacemaker implantation for AVB, challenging the assumption that statins may prevent AF in this population.

3. Survival benefits: While statin use did not improve atrial function or reduce AF incidence, it was associated with a significant reduction in all-cause mortality over five years. This highlights the benefit of statins in improving overall survival, although their effect on specific cardiac outcomes, like atrial function, remains limited.

4. Implications for future therapeutics: The results indicate that alternative therapeutic strategies are necessary to address atrial myopathy and fibrosis in patients with RV pacing-induced interventricular dyssynchrony, as statins do not provide significant protective effects in this area. Research into other anti-fibrotic or pacing optimization therapies may be needed.

## Supplementary Material

Supplementary figures and tables.

## Abbreviations

LA: Left atrial
AF: Atrial fibrillation
AVB: Atrioventricular block
RV: right ventricular
IPTW: inverse probability of treatment weighting
PPM: Permanent pacemaker: GSK-3β: Glycogen synthase kinase-3 beta
RVA: RV pacing plus atorvastatin
VDD: ventricular pacing and dual sensing
LAEF: Left atrial ejection fraction
ESA: End-systolic area
ESV: End-systolic volume
EDV: End-diastolic volume
LiCl: Lithium Chloride
CGRD: Chang Gung Research Database
CV: cardiovascular
LVEF: Left ventricular ejection fraction
LVEDD: Left ventricular end-diastolic diameter
HR: Hazard ratio
CI: confidence interval
p-GSK: GSK phosphorylation
ASMD: Absolute standardized mean differences
CABG: Coronary artery bypass graft
SIRT-1: Sirtuin 1
CTL: Control
GAPDH: Glyceraldehyde 3-phosphate dehydrogenase
