# Supplementary material for: Statins and Pacing-Induced Atrial Myopathy: Unraveling Their Role in Atrioventricular Block Patients with Permanent Pacemakers
Source: Int J Med Sci. 2025 Jul 19;22(13):3342–53. doi: 10.7150/ijms.113087 (PMC12320783; doi:10.7150/ijms.113087)
Supplement: Supplementary file 1 — Supplementary figures and tables. [file ijmsv22p3342s1.pdf]

Table S1. Baseline characteristics for unweighted cohort and Inverse Probability of Treatment-Weighted (IPTW) cohort

	Unweighted cohort, No. (%)			IPTW[1] cohort, %		
**Variables**	Statin	Non-statin	ASMD	Statin	Non-statin	ASMD
**Total**	304	2034				
**Age, years (mean, sd)**	74.6 (9.6)	72.9 (14.6)	0.139	74.6 (9.6)	74.9 (4.0)	0.037
**Age group**						
<65 years	46 (15.1)	447 (22.0)	0.177	46 (15.1)	40 (13.1)	0.059
65-74 years	103 (33.9)	501 (24.6)	0.204	103 (33.9)	104 (34.3)	0.009
≥75 years	155 (51.0)	1086 (53.4)	0.048	155 (51.0)	160 (52.6)	0.032
**Male gender**	159 (52.3)	1026 (50.4)	0.037	159 (52.3)	171 (56.4)	0.082
**Comorbidities**						
Hypertension	270 (88.8)	1423 (70.0)	0.479	270 (88.8)	268 (88.3)	0.082
Diabetes mellitus	189 (62.2)	690 (33.9)	0.589	189 (62.2)	183 (60.3)	0.038
Dyslipidemia	195 (64.1)	419 (20.6)	0.982	195 (64.1)	193 (63.6)	0.011
Gout	55 (18.1)	171 (8.4)	0.289	55 (18.1)	60 (19.8)	0.044
COPD	57 (18.8)	295 (14.5)	0.114	57 (18.8)	51 (16.8)	0.051
Peripheral arterial disease	13 (4.3)	60 (3.0)	0.071	13 (4.3)	13 (4.3)	<0.001
Chronic kidney disease	134 (44.1)	490 (24.1)	0.431	134 (44.1)	138 (45.3)	0.025
Dialysis	3 (1.0)	39 (1.9)	0.078	3 (1.0)	4 (1.4)	0.034
**Medications**						
ACEi/ARB	206 (67.8)	445 (21.9)	1.040	206 (67.8)	209 (68.8)	0.023
Beta blocker	83 (27.3)	185 (9.1)	0.486	83 (27.3)	87 (28.7)	0.030
DCCB	85 (28.0)	256 (12.6)	0.390	85 (28.0)	87 (28.6)	0.014
NDCCB	18 (5.9)	33 (1.6)	0.227	18 (5.9)	18 (5.9)	<0.001
Digoxin	4 (1.3)	14 (0.7)	0.063	4 (1.3)	5 (1.8)	0.038
Spironolactone	19 (6.3)	25 (1.2)	0.267	19 (6.3)	18 (5.8)	0.019
Metformin	64 (21.1)	59 (2.9)	0.582	64 (21.1)	53 (17.3)	0.095
DPP4i	35 (11.5)	37 (1.8)	0.396	35 (11.5)	32 (10.4)	0.034
Sulfonylurea	54 (17.8)	83 (4.1)	0.450	54 (17.8)	60 (19.9)	0.054
Thiazolidinedione	8 (2.6)	10 (0.5)	0.173	8 (2.6)	13 (4.3)	0.091
Insulin	21 (6.9)	16 (0.8)	0.322	21 (6.9)	16 (5.4)	0.064

**Baseline Lab data (mean, sd)[2]**						
Glycohemoglobin (%)	7.2 (1.6)	6.8 (1.3)	0.327	7.2 (1.6)	7.1 (0.8)	
Hematocrit (%)	34.6 (6.4)	34.0 (6.1)	0.098	34.6 (6.4)	33.2 (2.5)	
HDL (mg/dl)	47.8 (13.8)	44.7 (13.4)	0.232	47.8 (13.8)	45.0 (6.9)	
LDL (mg/dl)	93.1 (37.0)	98.6 (36.5)	0.148	93.1 (37.0)	103.9 (23.7)	
Total CHOL (mg/dl)	169.1 (42.1)	167.1 (38.8)	0.049	169.1 (42.1)	173.2 (20.9)	
eGFR (ml/min/1.73 m^2)	59.0 (30.3)	63.9 (37.7)	0.144	59.0 (30.3)	54.4 (16.0)	
**Echocardiography (mean, sd)[2]**						
Peri-implanted period						
LVEF (%)	66.0 (14.7)	66.6 (12.0)	0.044	66.0 (14.7)	66.7 (4.7)	
LVEDD (mm)	49.1 (8.2)	48.1 (7.2)	0.133	49.1 (8.2)	48.8 (2.7)	
LA (mm)	39.5 (6.6)	38.1 (7.0)	0.201	39.5 (6.6)	38.8 (2.6)	
**Outcome**						
Heart failure admission	20 (6.6)	117 (5.8)	0.034	20 (6.6)	19 (6.3)	0.012
Atrial fibrillation	32 (10.5)	229 (11.3)	0.024	32 (10.5)	32 (10.6)	0.001
Cardiovascular death	35 (11.5)	309 (15.2)	0.108	35 (11.5)	51 (16.7)	0.150
All-cause mortality	113 (37.2)	1003 (49.3)	0.247	113 (37.2)	164 (54.0)	0.342

Data are presented as mean ± SD or number (percentage).

Abbreviations: ACEi/ARB: angiotensin-converting enzyme inhibitors/angiotensin II receptor blockers; COPD: chronic obstructive pulmonary disease; CKD: chronic kidney disease; DCCB: Dihydropyridine calcium channel blocker; DPP4i: Dipeptidyl peptidase 4 inhibitors. HDL: high density lipoprotein; IPTW: inverse probability of treatment-weighted; LDL: low density lipoprotein; LVEDD: left ventricular end-diastolic diameter; LVEF: left ventricular ejection fraction; NDCCB: Non-dihydropyridine calcium channel blocker; PAD: peripheral artery disease

[1]IPTW adjustment on age, gender, comorbidities and medications.

[2]Maximum effective sample size.

Table S2. Hazard ratio for study outcomes between statin and non- statin groups by different analysis approaches

	Unweighted Sample				IPTW-ATT			
	Univariate		Multivariate[1]		Univariate		Multivariate[1]	
	HR (95% CI)	*P*-value	HR (95% CI)	*P*-value	HR (95% CI)	*P*-value	HR (95% CI)	*P*-value
**Heart failure admission**								
1 year	0.90 (0.35-2.30)	0.824	1.17 (0.43-3.20)	0.766	1.89 (0.87-4.07)	0.106	1.88 (0.87-4.06)	0.111
3 years	1.47 (0.78-2.77)	0.230	1.67 (0.83-3.35)	0.151	1.19 (0.77-1.85)	0.437	1.28 (0.82-1.99)	0.282
5 years	1.10 (0.63-1.91)	0.735	1.20 (0.66-2.18)	0.561	0.91 (0.63-1.32)	0.627	0.98 (0.68-1.42)	0.913
**Atrial fibrillation**								
1 year	0.67 (0.34-1.34)	0.257	0.65 (0.31-1.34)	0.238	0.71 (0.46-1.10)	0.128	0.72 (0.46-1.12)	0.141
3 years	0.99 (0.63-1.57)	0.970	0.95 (0.58-1.56)	0.830	1.01 (0.74-1.39)	0.948	1.02 (0.74-1.41)	0.885
5 years	0.99 (0.66-1.48)	0.962	0.98 (0.63-1.51)	0.911	1.11 (0.84-1.48)	0.463	1.11 (0.83-1.48)	0.475
**Cardiovascular mortality**								
1 year	0.50 (0.20-1.24)	0.135	0.42 (0.16-1.07)	0.070	0.49 (0.28-0.85)	0.011	0.46 (0.27-0.80)	0.006
3 years	0.47 (0.25-0.90)	0.023	0.41 (0.21-0.81)	0.010	0.46 (0.31-0.68)	<0.001	0.45 (0.30-0.66)	<0.001
5 years	0.57 (0.35-0.94)	0.027	0.51 (0.30-0.87)	0.012	0.58 (0.43-0.79)	<0.001	0.57 (0.42-0.78)	<0.001
**All-cause mortality**								
1 year	0.36 (0.19-0.68)	0.002	0.29 (0.15-0.56)	<0.001	0.48 (0.33-0.71)	<0.001	0.47 (0.32-0.70)	<0.001
3 years	0.54 (0.38-0.76)	<0.001	0.45 (0.31-0.65)	<0.001	0.52 (0.42-0.64)	<0.001	0.51 (0.41-0.63)	<0.001
5 years	0.61 (0.46-0.80)	<0.001	0.54 (0.41-0.72)	<0.001	0.58 (0.49-0.69)	<0.001	0.58 (0.49-0.69)	<0.001

Abbreviations: IPTW: inverse probability of treatment-weighted; HR: hazard ratio; CI: confidence interval.

[1]Models were adjusted by sex, age group, hypertension, diabetes mellitus, dyslipidemia, gout, COPD and chronic kidney disease.

Table S3. Comparative Analysis of LA Dimension, LVEF, and LVEDD Between Statin and Non-Statin Groups Over a 5-Year Follow-Up Period

	**Unweighted cohort**							**IPTW[1] cohort**						
	Statin			Non-statin				Statin			Non-statin			
**Follow years**	N	Mean	SD	N	Mean	SD	P value	N	Mean	SD	N	Mean	SD	P value
Peri-implanted period														
LA (mm)	232	39.5	6.6	1462	38.1	7.0	0.006	232	39.5	6.6	1462	38.8	2.6	0.128
LVEF (%)	252	66.0	14.7	1535	66.6	12.0	0.545	252	66.0	14.7	1535	66.7	4.7	0.508
LVEDD (mm)	253	49.1	8.2	1541	48.1	7.2	0.061	253	49.1	8.2	1541	48.8	2.7	0.542
1 Year														
LA (mm)	86	40.9	7.1	349	39.3	7.9	0.079	86	40.9	7.1	349	40.3	2.9	0.486
LVEF (%)	94	58.0	18.0	407	61.3	15.1	0.100	94	58.0	18.0	407	59.0	9.5	0.649
LVEDD (mm)	97	51.3	10.0	409	49.4	8.8	0.063	97	51.3	10.0	409	48.7	3.2	0.016
3 Years														
LA (mm)	64	40.5	7.6	266	39.2	7.5	0.210	64	40.5	7.6	266	39.5	2.5	0.334
LVEF (%)	78	60.7	15.4	291	61.5	14.5	0.668	78	60.7	15.4	291	59.0	6.0	0.388
LVEDD (mm)	78	48.7	7.3	294	48.7	7.8	0.963	78	48.7	7.3	294	50.0	3.1	0.176
5 Years														
LA (mm)	42	39.5	8.1	170	38.3	7.9	0.388	42	39.5	8.1	170	37.9	2.7	0.264
LVEF (%)	48	56.7	18.2	192	60.1	15.1	0.189	48	56.7	18.2	192	58.9	5.2	0.462
LVEDD (mm)	49	49.5	8.1	194	49.2	8.1	0.826	49	49.5	8.1	194	49.6	2.8	0.902

Data are presented as mean ± SD.

Abbreviations: IPTW: inverse probability of treatment-weighted; LA: left atrium; LVEF: left ventricular ejection fraction; LVEDD: left ventricular end-diastolic diameter.

Supplemental Figure legends

Supplemental Figure 1. Study Design Flowchart for the Evaluation of the Effects of Pacing and Atorvastatin in Lanyu Miniature Pigs

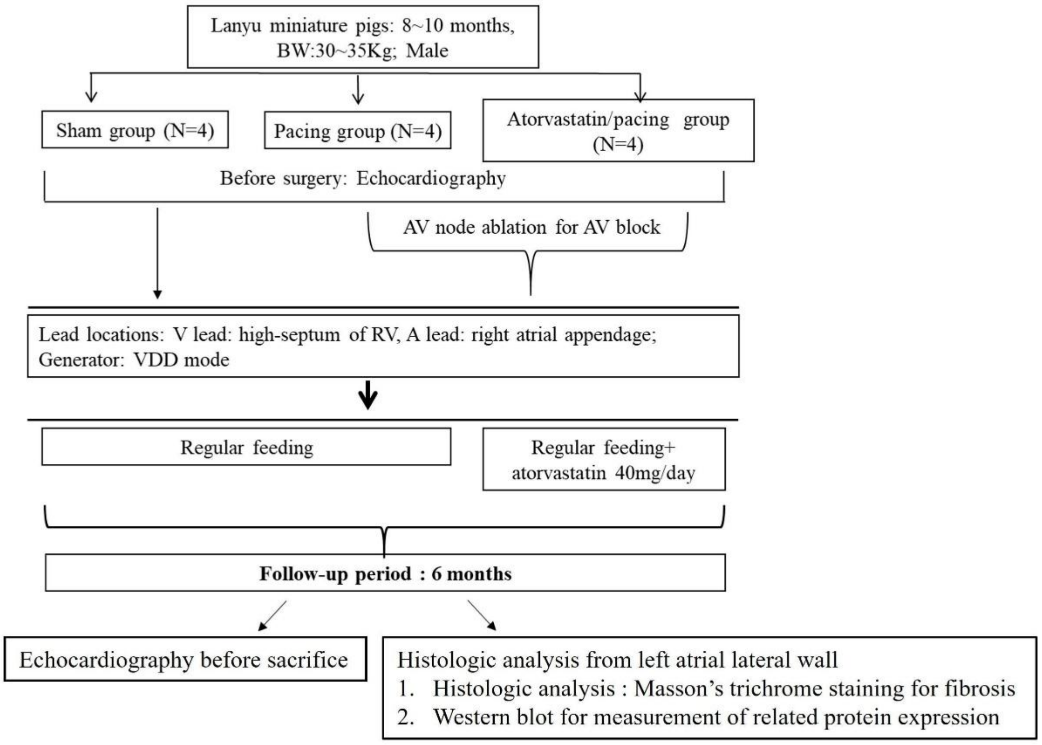

This flowchart illustrates the experimental design involving Lanyu miniature pigs (8-10 months old, body weight: 30-35 kg, male) divided into three groups:

- Sham group (N = 4): Underwent echocardiography before surgery but no further intervention.
- Pacing group (N = 4): Underwent echocardiography before surgery, followed by atrioventricular (AV) node ablation to induce AV block and the implantation of pacemaker leads (ventricular lead at the high septum of the right ventricle and atrial lead in the right atrial appendage) with VDD mode pacing.
- Atorvastatin/pacing group (N = 4): Similar procedure as the pacing group but received daily atorvastatin (40 mg/day) during the 6-month follow-up period.

Abbreviations: BW: body weight; AV: atrioventricular; VDD: ventricular pacing and

dual sensing.

Supplemental Figure 2. Catheter Ablation for the Creation of Atrioventricular Block in the Animal Model: Fluoroscopic Imaging and Electrogram Signals

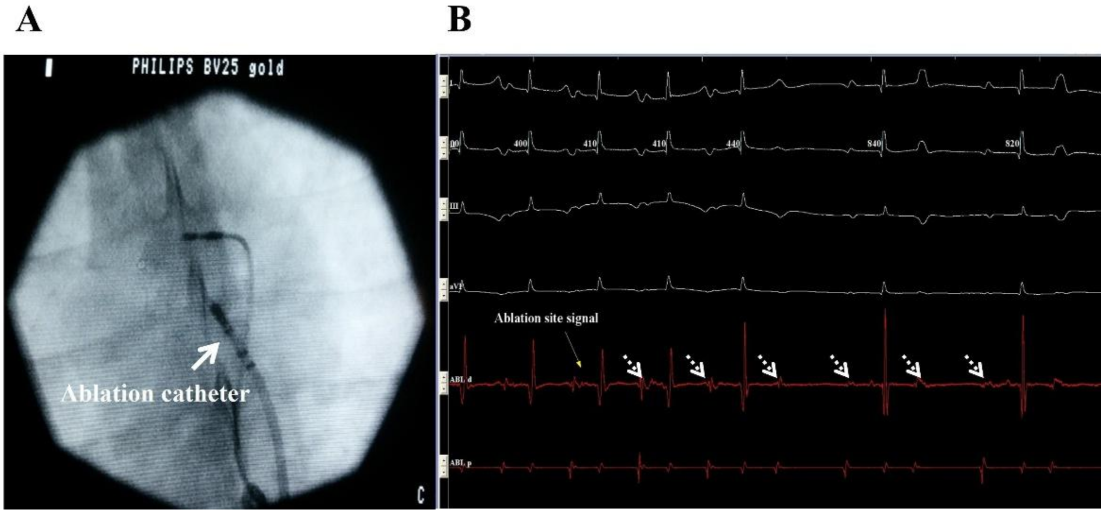

(A) Fluoroscopic image: The position of the ablation catheter (white arrow) presented during the creation of atrioventricular block procedure.

(B) Electrogram recordings at the ablation site: The red tracing highlights the ablation site signal (left white arrow), showing consistent local electrogram activity that precedes the atrial signal but is not followed by a ventricular signal (indicated by the right white arrows), confirming atrioventricular dissociation.

Supplemental Figure 3. Experimental Design for HL-1 Atrial Myocyte Stretching Model

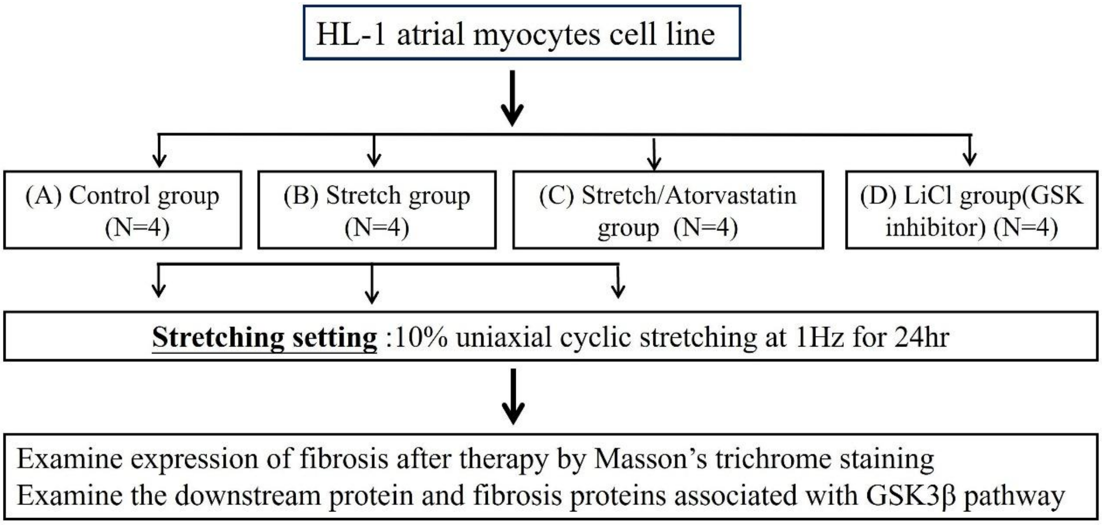

This flowchart illustrates the experimental design using HL-1 atrial myocyte cell lines divided into three groups:

- (A) Placebo group (N = 4): Cells were cultured without mechanical stretching or drug treatment.
- (B) Stretching group (N = 4): Cells underwent 10% uniaxial cyclic stretching at a frequency of 1 Hz for 24 hours.
- (C) Stretching/Atorvastatin group (N = 4): Cells were subjected to the same stretching conditions as the stretching group and were also treated with atorvastatin.
- (D) LiCl group (N = 4): Cells were subjected to the same stretching conditions as the stretching group and were also treated with LiCl.

Abbreviations: GSK: glycogen synthase kinase; LiCl: Lithium Chloride; N: Number.

Supplemental Figure 4. Patient Selection Flowchart for the Study of Statin Use in Atrioventricular Block (AVB) Patients Undergoing Permanent Pacemaker (PPM) Therapy

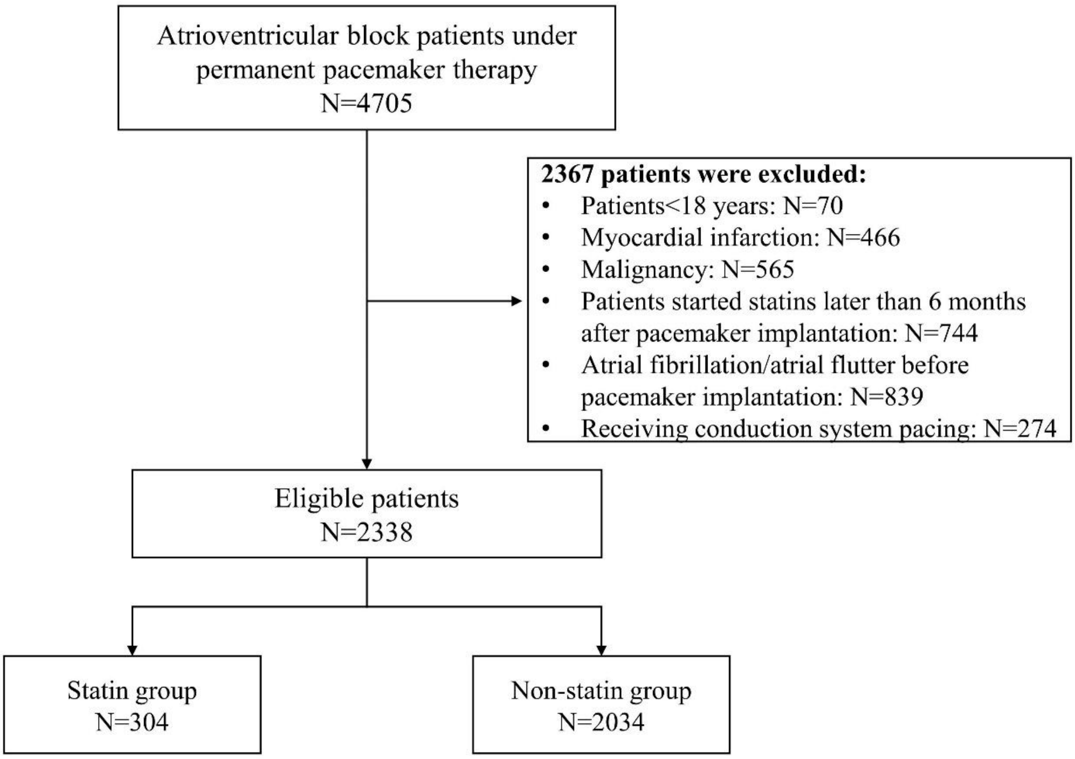

This flowchart depicts the process of selecting eligible patients from an initial cohort of 4705 AVB patients under PPM therapy. The following exclusion criteria were applied to refine the cohort:

- Exclusions (N = 2338):
  - Patients under 18 years of age (N = 70)
  - Patients with a history of myocardial infarction (N = 466)
  - Patients with malignancies (N = 565)
  - Patients who started taking statins more than 6 months after PPM implantation (N = 744)
  - Patients with atrial fibrillation or atrial flutter (N = 839)
  - Patients receiving conduction system pacing (N = 274)

After applying these exclusions, a total of 2338 eligible patients remained, who were then divided into two groups:

- Statin group (N = 304): Patients who were on statin therapy.
- Non-statin group (N = 2034): Patients who were not on statin therapy.

Abbreviations: AVB: atrioventricular block; PPM: permanent pacemaker; N: Number.

Supplemental Figure 5. Comparison of Heart Rate (Bpm) Between Sham Control, Pacing, and Atorvastatin/Pacing Groups

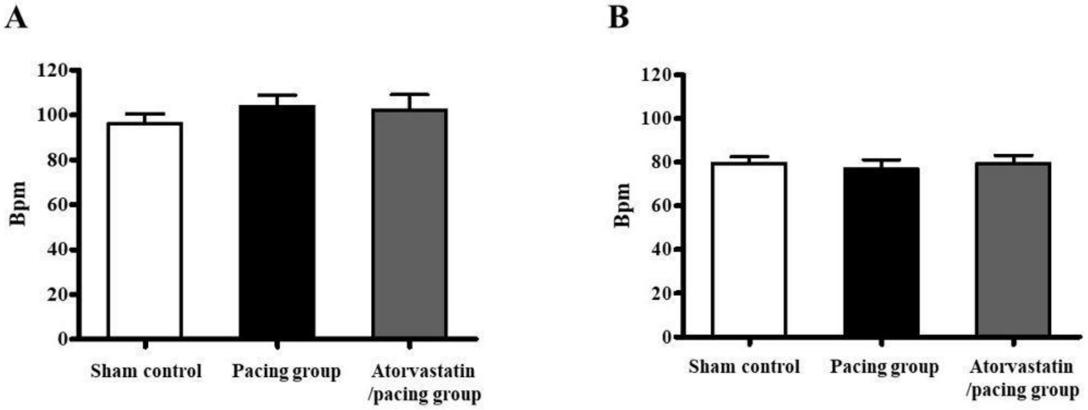

(A) Baseline heart rate (beats per minute, Bpm) measured before the start of the intervention. No significant differences in heart rate are observed between the sham control, pacing, and atorvastatin/pacing groups, indicating similar baseline conditions. (B) Heart rate measured before sacrifice. No significant differences in heart rate are observed between the three groups, suggesting that pacing and atorvastatin treatment did not significantly alter heart rate before sacrifice.

Abbreviations: Bpm: beats per minute.

Supplemental Methods

Masson's trichrome staining

Sections of the lateral wall of LA myocardium were stained and analyzed using a modified Masson’s trichrome stain kit (ScyTek Laboratories, Logan, UT, USA) following the manufacturer’s protocol. Briefly, 5-μm sections were deparaffinized, fixed with Bouin’s solution, and stained with Weigert’s iron hematoxylin, followed by Biebrich scarlet/acid fuchsin in a phosphomolybdic/phosphotungstic acid solution, and then aniline blue with acetic acid. After dehydration, sections were mounted and visualized using an Olympus DP70 microscope. Fibrosis was quantified as the percentage of positively stained areas using Image Pro Plus software (version 6.0; Media Cybernetics, Silver Spring, MD, USA).

Western blotting

Protein extracts from left atrial (LA) tissues were prepared using CelLytic™ MT Cell Lysis Reagent (Sigma-Aldrich, St. Louis, MO, USA). Homogenates were centrifuged at 14,000 rpm for 30 min at 4 °C to obtain supernatants, and protein concentrations were measured using the Bradford method (Bio-Rad, Hercules, CA, USA). Protein samples (30 μg) were separated on 10%–15% SDS-PAGE gels at room temperature for 1 h and transferred to PVDF membranes for 1.5 h on ice. Membranes were blocked with TBST containing 5% nonfat dry milk or 2% bovine serum albumin (BSA) at room temperature for 1 h. Primary antibodies, including collagen III (1:1000, Proteintech, TX, USA), p-GSK3β (Ser9), and total GSK3β (both 1:1000, Cell Signaling, MA, USA), were incubated overnight at 4 °C in TBST with 5% milk or 2% BSA. After three washes with TBST, membranes were incubated with HRP-conjugated secondary antibodies (1:5000) for 1 h at room temperature, followed by three additional washes. Signal detection was performed using Immobilon Western

HRP substrate (Millipore, Burlington, MA, USA). Protein levels were normalized to GAPDH (1:1000, Proteintech, TX, USA), and chemiluminescence was quantified using a BioSpectrum 810 imaging system (Analytik Jena, Germany).
